# Selection of appropriate reference genes for quantitative real-time PCR in *Oxytropis ochrocephala* Bunge using transcriptome datasets under abiotic stress treatments

**DOI:** 10.3389/fpls.2015.00475

**Published:** 2015-06-30

**Authors:** Huihui Zhuang, Yanping Fu, Wei He, Lin Wang, Yahui Wei

**Affiliations:** Key Laboratory of Resource Biology and Biotechnology in Western China, Department of Life Science, Northwest UniversityXi'an, China

**Keywords:** *Oxytropis ochrocephala* Bunge, reference genes, quantitative real-time PCR, transcriptome, abiotic stress

## Abstract

**Background:** *Oxytropis ochrocephala* Bunge, an indigenous locoweed species in China, poses great threats to livestock on grasslands. There is a need for further genetic study in the plants *per se*, for understanding the basis of its acclimation mechanism in various unfavorable environmental conditions and to implement effective control measures. Quantitative real-time reverse transcription-polymerase chain reaction (qRT-PCR) is the most commonly used method for gene expression analysis. To facilitate gene expression studies and obtain more accurate qRT-PCR data, normalization relative to stable reference genes is required. The aim of this study was to select the most stable reference genes for transcriptional analysis in *O. ochrocephala*.

**Results:** We selected 12 candidate reference genes, 18S ribosomal RNA (*18S RNA*), actin2/7 (*ACT7*), β-actin (*ACTB*), actin101 (*ACT101*), actin11 (*ACT11*), β-tubulin (*TUB*), α-tubulin (*TUA*), glyceraldehyde-3-phosphate dehydrogenase-1 (*GAPDH1*), *GAPDH2*, metallothionein-like protein (*MET*), fructose-bisphosphate aldolase (*FBA*) and histone H3 (*HIS*), from the transcriptome datasets of *O. ochrocephala* and determined the suitability by analyzing their expression levels when exposed to a range of abiotic stress conditions. By employing software packages including geNorm, NormFinder and BestKeeper, *HIS, ACT7*, and *ACT101* were assessed as the most suitable set for normalization in all samples. When normalized with the most stable reference genes, the expression patterns of the three target genes were in accordance with those in the transcriptome data, indicating that the reference genes selected in this study are suitable.

**Conclusions:** The study provided appropriate reference genes for accurate normalization in qRT-PCR analysis in *O. ochrocephala* and emphasized the importance of validating reference genes for gene expression analysis under specific experimental condition. The usage of inappropriate reference gene would cause misinterpretation.

## Introduction

*Oxytropis ochrocephala* Bunge (Fabaceae), a perennial grass species, grows aggressively on the grassland of northwestern China. It is the most widespread locoweed (the collective name for toxic *Oxytropis* and *Astragalus* species) in China. Locoweeds contain an indolizidine alkaloid swainsonine, an α-mannosidase inhibitor which causes over-accumulation of mannose rich oligosaccharide in the lysosomes and impairs the neural system of livestock, which has long been recognized as the principle for intoxication in animals (Ralphs et al., 2008). Although it has been proved that swainsonine is produced by its symbiotic fungal endophytes, the plants *per se* also have a great impact on the production of swainsonine (Pryor et al., 2009). Cook et al. (2013) observed that plants offer the endophytes nutrients, hormones and some other signals in influencing its capacity to produce the toxin. In addition, *O. ochrocephala* grows in high-altitude areas with deficient water, high soil saline content, and extreme low environmental temperature, indicating that the plant has the ability to cope with unfavorable environmental stresses. In order to better understand the role of the plant in production of swainsonine, the research on its stress response mechanism to harsh environment conditions is needed. Currently, genetic information of locoweeds for molecular biology research is limited in the public databases, which makes it more difficult for further in-depth research. A recently EST dataset of a suppressive subtraction cDNA library enriched in genes from two temperate *Oxytropis* species was made available in NCBI, but it only has 1245 ESTs and thus there is a need for further exploring the genetic information in *Oxytropis* (Chung et al., 2004; Archambault and Strömvik, 2011).

RNA sequencing (RNA-Seq), with the rapid development of the next-generation sequencing technology, has been applied prevalently on analyzing the transcriptomes of various species for a range of purposes (Wang et al., 2009; Li et al., 2015a; Stone and Storchova, 2015). The main outcome of RNA-Seq data is the identification of differentially expressed genes, while it was also used to search for reference genes. Any gene with a minimal expression level variation in every analyzed sample is considered as candidate reference gene. Meanwhile, qRT-PCR is commonly used to determine expression levels of genes and to validate transcriptomic data (Andersen et al., 2004; Caldana et al., 2007). For accurate qRT-PCR evaluation, it is necessary to select suitable reference genes as internal control under different experimental conditions because the starting material, RNA extraction, RT-PCR efficiency, and qRT-PCR efficiency can vary among experiments. In addition, gene expression can be highly tissue-specific and often varies based on the physiological status of the organism or experimental treatments. Thus, it is essential for an optimal reference gene or combination of genes for the specific experiment to normalize gene expression data.

The growing acquisition of plant genomes and transcriptomes provide a high-throughput approach to identify a set of reference genes. In the study of Czechowski et al. (2005), based on the Arabidopsis genome, hundreds of genes with stable expression levels were identified. Yang et al. (2014b) used a similar approach to mine 40 genes as candidate reference genes from the transcriptome datasets of *Brassica napus* and 14 reference genes were selected for further analysis with qRT-PCR in different tissues and under different experimental treatments. Reference genes are usually cellular maintenance genes, which regulate basic cellular functions and components, such as *Actin* (Nakayama et al., 2014), *Tubulin* (Zhang et al., 2013), *GAPDH* (Ling et al., 2014), *18S RNA* (Yang et al., 2014a), *Ubiquitin* (*UBQ*) (Galeano et al., 2014), *Histone* (Wang et al., 2014; Yan et al., 2014). Recent studies have indicated that there is no universal reference gene for all experimental conditions and systematic expression stability analysis of reference genes under specific experimental conditions is necessary (Jian et al., 2008).

A few statistical algorithms have been developed to identify which reference gene(s) is (are) suitable for normalization under a given experiment condition for a specific species. These programs have been successfully employed to determine the stability of reference gene expression and identify stable reference genes for various plant species, such as tall fescue (Yang et al., 2015), moss (Li et al., 2015b), switchgrass (Gimeno et al., 2014), and *Caragana korshinskii* (Yang et al., 2014c). Because of the different algorithms, the rankings provided by different programs are often not completely identical, and Jacob et al. (2013) recommend that more than two algorithms should be used for reference gene stability evaluation.

In this study, we selected 12 candidate reference genes based on the abiotic transcriptome datasets of *O. ochrocephala* by RNA-Seq (unpublished data) and further validated the expression stability of these genes by qRT-PCR and evaluated them using popular software packages including geNorm (Vandesompele et al., 2002), NormFinder (Andersen et al., 2004) and BestKeeper (Pfaffl et al., 2004). Three target genes, 1-aminocyclopropane-1-carboxylate oxidase (*ACO*), calcium binding protein (*CaBP*) and cinnamate-4-monooxygenase (*C4H*), were used to validate the effectiveness of the selected reference genes. The three genes *ACO, CaBP*, and *C4H* represent the characteristics of stress-responsive genes in plants. Under drought stress, *ACO* gene is up-regulated to increase the production of ethylene which is a key plant hormone to regulate plant growth and development (Chen et al., 2005). *CaBP* gene is a member of the calcium signaling system, which regulates an important pathway in plant cold acclimation (Yang et al., 2010). *C4H* is involved in the accumulation of soluble phenylpropanoids which act as the radical scavenger, helping to reduce cell oxidative damage caused by biotic and abiotic stress (Hemm et al., 2004). This is the first report which has determined the reference genes in *O. ochrocephala* and the other *Oxytropis* species. In particular, this work provides the basis for further research in exploring the mechanism of adaption *O. ochrocephala* to stress environments.

## Materials and methods

### Plant materials and stress treatments

Seeds of *O. ochrocephala* were collected from Haiyuan, Ningxia province. Seeds were pre-treated with concentrated sulphuric acid for 8 min, then washed three times with distilled water and placed onto wet filter papers in Petri dishes for germination. Germinated seeds were kept growing until cotyledons emerged and then transferred into individual pots (5 × 5 × 6 cm) filled with sand and peat (1:1), and grown in the growth chamber under controlled conditions at a photo flux density of 300 μmol m^−2^s^−1^ (14/10 h, day/night period), at a relative humidity of 55–60%, and a temperature of 25 ± 2°C (Tang et al., 2007). Each group of 10 pots of 4-week-old seedlings with consensus growth status were selected and treated by drought, cold and salt stress, relatively. Three biological replicates were included for each treatment and control.

For drought treatment, 20% PEG-6000 solution (w/v, polyethylene glycol, Sangon, China) was applied to irrigate the plants in the soil every 4 h, simulating osmotic stress which is equivalent to −0.54 MPa, according to Jones and Gorham (1983); For cold treatment, seedlings were transferred into another growth chamber at 4°C with the rest growing conditions being the same. For salt treatment, 150 mM NaCl (Sangon, China) solution was applied to irrigate the plants. Entire seedlings were collected at different times (0, 3, 6, 12 h) from the onset of treatment and immediately frozen in liquid nitrogen for RNA extraction.

### Total RNA extraction and cDNA synthesis

Total RNA was extracted according to the modified CTAB method (Gasic et al., 2004). To eliminate DNA contamination, total RNA was DNase I (Ambion, USA) treated and purified according to the manufacturer's protocol. The integrity of total RNA samples were verified by 1.2% (w/v) agarose gel electrophoresis, and the quantity and quality of RNA samples were measured with the NanoDrop ND-1000 Spectrophotometer (NanoDrop Technologies, USA).

In order to perform qRT-PCR, cDNA was synthesized by reverse transcription using 2.5 μg total RNA in a 20 μl reaction volume according to the manufacturer's instructions (Thermo Scientific, USA). The cDNA was diluted 10-fold with nuclease-free water for qRT-PCR.

### Transcriptome data mining for candidate reference genes

We performed transcriptome sequencing of *O. ochrocephala* using Illumina paired-end sequencing technology on an Illumina Hi-Seq™ 2000 platform for the four samples (control, drought, cold and salt). After assembly and annotation, a software package, RSEM (RNA-Seq by Expectation Maximization) (Li and Dewey, 2011), was employed to analyze the read counts, which were then converted to fragments Kilobase of exon model per millon mapped reads (FPKM values), a commonly accepted estimate for the expression level of unigenes (Trapnell et al., 2010). To estimate expression stability of each gene, the following indices of FPKM values, including mean expression value (MV), standard deviation (SD) and coefficient of variation (CV) value (dividing SD by MV) were calculated according to the methodology described by de Jonge et al. (2007). Genes with lower CV values were considered to be stably expressed and the cut-off CV value was set as lower than 0.3, suggested by Czechowski et al. (2005) and Narsai et al. (2010).

Twelve candidate reference genes were selected based on gene annotation to NR database. Sequences of the candidate reference genes were used to design primers using Primer5 software. The criteria for primer design were set as follows: melting temperature (T_m_) in a range of 55–60°C, primer lengths of 18–24 bp, GC contents of 45–55% and amplicon lengths of 95–282 bp.

### RT-PCR and qRT-PCR analysis

To verify the specificity of all the primer sets, PCR was performed using pooled cDNA as templates, and the PCR products were examined by 2% (w/v) agarose gel electrophoresis. The amplicons should appear as a single band with the correct size. To confirm that the PCR product did not contain multiple amplicons of the same size, which were amplified from possibly existing several genes in a gene family, eight individual PCRs were performed for each gene and the products were sequenced. Then the genes were further analyzed by qRT-PCR.

qRT-PCR reactions were performed in 20 μl system on a Bio-Rad CFX96 Real-Time PCR system (Bio-Rad, USA) with a reaction contained 10 μl of FastStart Universal SYBR GreenMaster (Roche, Germany), 2 μl of diluted cDNA template, 1 μl of each primer (10 μM). For each gene, a no template control (NTC) was included using water instead of cDNA as template. The qRT-PCR reactions were conducted following the fast thermal cycles: 95°C for 10 min, 40 cycles at 95°C for 30 s, 58°C for 30 s, and 72°C for 30 s. After 40 cycles, the dissociation curve was performed to confirm the specificity of each primer again by heating up the product from 60°C to 95°C. The Rn (normalized reporter) threshold was automatically selected to obtain the cycle threshold (Ct) values. The final Ct value of each sample was the mean of three biological replicates and three technical replicates. The mean amplification efficiency of each primer pair was checked by the LinRegPCR program (Ruijter et al., 2009).

### Data analysis of gene expression stability

Three different types of programs, geNorm (version 3.5), BestKeeper and NormFinder were used to rank the stability of the selected reference genes across all the experimental sets (Xia et al., 2014; Yang et al., 2014c; Galli et al., 2015). For geNorm and NormFinder, the raw Ct value of each gene was converted into the relative quantities by the formula 2^−ΔCt^ (ΔCt = each corresponding Ct value - lowest Ct value). For BestKeeper, the Ct value was used to calculate the standard deviation (SD) and the coefficient of variation (CV). The most stable gene exhibits the lowest CV ± SD value. Genes with SD value greater than 1 were considered to be unacceptable as reference genes. The pairwise variation (Vn/Vn+1) between two sequential normalization factors was obtained by geNorm software for determine the optimal number of reference genes needed to normalize. The recommended cut-off threshold is 0.15, below which an additional control gene is not required for normalization (Vandesompele et al., 2002). The overall recommended rankings of the best reference genes were obtained using the results of three algorithms.

### Validation of reference genes

The expression pattern of three target genes *ACO*, *CaBP*, and *C4H* were analyzed using the most stable and least stable reference genes after normalization across three experimental sets, drought stress (for *ACO*), cold stress (for *CaBP*) and salt stress (for *C4H*). To validate the results, the expression levels of the target genes in qRT-PCR were compared with the FPKM values in RNA-seq data in each sample (Figure S2). The sequences of these genes were obtained from the transcriptome data (Table S3). The amplification efficiencies of the target genes were also estimated by the LinRegPCR program. The average Ct value was calculated from three biological and technical replicates and used for relative expression analyses. The relative expression data were calculated according to the 2^−ΔΔCT^ method and presented as fold change (Livak and Schmittgen, 2001).

## Results

### Identification of putative reference genes based on transcriptome datasets

Based on the FPKM value, we selected 12 genes with low CV values (below 0.3) (*18S*, *ACT101, ACTB, ACT7, ACT11*, *GAPDH1, GAPDH2*, *TUB*, *TUA*, *MET*, *FBA*, and *HIS*) and these genes have been reported as stable genes in other species. The sequences of the 12 reference genes were obtained from the transcriptome data. Gene symbol, gene length and function description were shown in Table 1. We used full length unigene sequences from transcriptome data to design the specific primers for qRT-PCR. Primer pairs were designed to avoid the conserved domains or at the untranslated region (UTR) of each gene. For the amplified sequences of these homolog genes, nucleotide identities were relatively lower, around 30–50% range. Amino acid identities among homologous genes were less than 30%, suggesting less functional identity of the amplified fragment (Table S1).

**Table 1:** **Description of the candidate reference genes in transcriptome datasets of *O. ochrocephala***.

**Gene symbol**	**Gene ID**	**Accession number**	**Gene length (bp)**	**NR description**	**NR accession numbers**	**CK_FPKM**	**PEG_FPKM**	**COLD_FPKM**	**SALT_FPKM**	**CV**
*18S*	comp82308_c0	KR822224	7829	ribosomal RNA gene [*Medicago truncatula*]	XP_003614382.1	702.31	619.06	650.75	642.99	0.054
*ACT101*	comp81236_c0	KR822225	1849	Actin 101 [*Glycine max*]	XP_003547582.1	343.95	343.4	395.6	321.39	0.089
*ACTB*	comp82568_c0	KR822226	732	Beta-actin [*Pisum sativum*]	AAB18643.1	29.9	28.47	21.23	20.88	0.188
*ACT7*	comp79791_c0	KR822227	1936	Actin 2/7 [*Glycine max*]	XP_003523242.1	409.7	433.17	541.08	414.85	0.137
*ACT11*	comp81236_c3	KR822228	1167	Actin 11 [*Medicago truncatula*]	XP_003622019.1	178.49	168.43	192.51	164.35	0.071
*TUA*	comp80981_c0	KR822229	1970	Alpha-tubulin [*Medicago truncatula*]	XP_003629736.1	204.66	175.09	156.08	156.78	0.132
*TUB*	comp49473_c0	KR822230	1969	Beta-Tubulin [*Medicago truncatula*]	XP_003603765.1	169	135.68	129.07	121.21	0.152
*GAPDH1*	comp83553_c0	KR822231	2197	GAPDH1 [*Medicago truncatula*]	XP_003603851.1	403.17	386.48	276.76	346.27	0.159
*GAPDH2*	comp90007_c0	KR822232	1731	GAPDH2 [*Medicago truncatula*]	XP_003601828.1	1094.27	1141.33	968.64	1140.51	0.075
*MET*	comp71233_c0	KR822233	661	metallothionein-like protein [*Glycine max*]	XP_003520973.1	1976.38	1789.19	2507.53	2023.19	0.148
*FBA*	comp67830_c0	KR822234	1851	Fructose-bisphosphate aldolase [*Glycine max*]	XP_003537836.1	1479.71	1708.19	1020.81	1267.67	0.215
*HIS*	comp67746_c0	KR733680	955	Histone H3 [*Zea mays*]	DAA45649.1	654.76	620.72	509.92	616.37	0.104

### Expression profiles of reference genes

The primer specificities were confirmed by agarose gel electrophoresis, sequencing and melting curves, which showed the single amplicon of the expected size and the single peak melting curves (Figure S1, sequencing result not shown). The qRT-PCR products ranged from 95 to 282 bp. The amplification efficiencies of these reference genes were spanning from 1.8 to 2, and the linear R^2^ (correlation coefficients) ranged from 0.995 to 0.999. The primer sequences and relevant amplification information were presented in Table 2.

**Table 2:** **List of primer sequences of the candidate reference genes and their amplification efficiencies**.

**Name**	**Forward primer sequence [5′–3′]**	**Reverse primer sequence [5′–3′]**	**Amplicon Size (bp)**	**Product TM^a^ (°C)**	**qRT-PCR Efficiency^b^**	***R*^2^**
*18S*	CACCTTACGAGGGTCACC	GGTTTAGACCGTCGTGAGAC	282	87.5–88	1.807	0.998
*ACT101*	GGGTGAATATGATGAATCTGG	GTCTGGCTTTCAAGGACATAA	232	79.5–80	1.901	0.997
*ACTB*	CGGCATCCATGAGACAAC	TCTGGTGGTGCTACAACCTT	186	81–81.5	1.987	0.995
*ACT7*	CTCCACCAGAGAGAAAGTACAGT	CCCAGAATATACCCTCCGAT	266	80–80.5	1.908	0.995
*ACT11*	TCCTTCTTTGCCTTCCATC	GACCTACAATGCTGGGAAAC	168	81.5–82	1.907	0.997
*TUA*	GAAAGATTATGAGGAGGTTGG	TAACAGTTTGAATGATGGAACTATA	260	77.5–78.5	1.834	0.997
*TUB*	TATGCTCCTTCTTGCCGTAT	CAATACAAAACGAATTGGTAGG	188	80–80.5	1.961	0.996
*GAPDH 1*	CACAAATACAAGGTTTCCATCT	CACTGGTTCTCCCCTCACA	211	80–80.5	1.920	0.998
*GAPDH 2*	TCTATTCTGGGTTGGAGTCTT	CCTTTGCTCTGCCTCACA	162	77.5–78	1.901	0.997
*MET*	TGTTTGGAGTTTTAGAATGAAGTG	TTCTATGCCAAGTAGATGATGG	251	75–76	1.910	0.997
*FBA*	GAATCATTTTCTTGCCAGTG	ATGAATCTCCTGCAACCTT	224	76–77	1.902	0.999
*HIS*	ATGGGCTTGGTGCTGTTA	CCTTCATCAGACTAGCAGTAAAC	95	76.5–77	2.047	0.997

a *The melting temperature was calculated by Bio-Rad CFX Manager software*.

b *The mean qRT-PCR amplification efficiency and correlation coefficients (R^2^) for each primer pair were determined by LinReg PCR software*.

**Figure 1:**
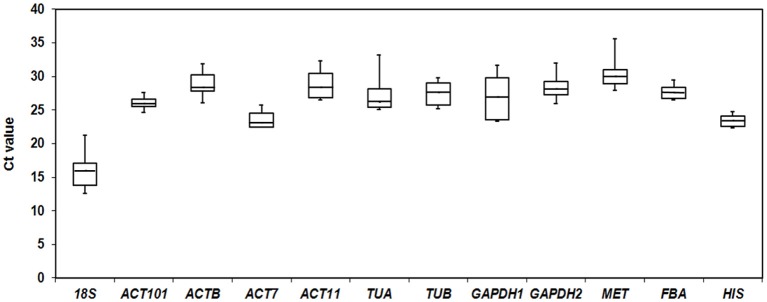
**Cycle threshold (Ct) values of 12 candidate reference genes across all samples**. The final Ct value of each sample was the mean of three biological and technical replicates. Box graph indicates the interquartile range. A line across the box showed as the median. Lower and upper dashes represent the maximum and minimum values.

To quantify overall differences in expression levels of the 12 reference genes, the median Ct value for each gene under total groups was calculated. The mean Ct values of the 12 candidate reference genes revealed a minimum of 15.93 ± 2.56 (*18S*) (mean ± SD) and a maximum of 30.34 ± 2.20 (*MET*) for highest and lowest expression levels, which showed the expression level of *MET* was almost 44-fold lower than *18S*. The genes with higher SD of Ct values indicated more variable expression compared to these with lower SD. *HIS* showed the smallest variation in gene expression (23.37 ± 0.79), while *18S* with the most variable levels of expression (Figure 1).

### geNorm analysis

**Figure 2:**
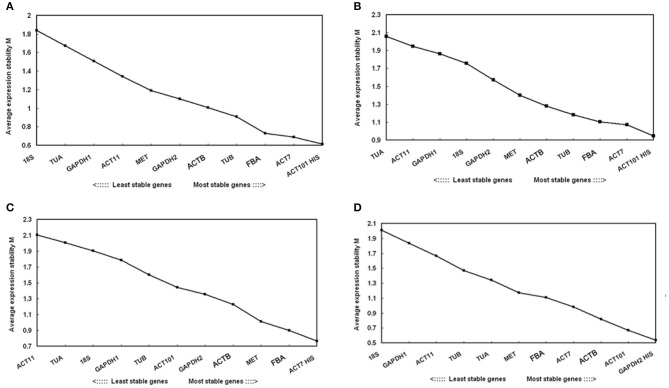
**Expression stability values (M) of 12 candidate reference genes calculated by geNorm**. Lower M values indicate more stable expression. Ranking of the gene expression stability was performed in all the samples and each abiotic stress samples. The least stable genes were on the left and the most stable genes on the right. **(A)** All tested samples **(B)** Drought stress **(C)** Cold stress **(D)** Salt stress.

Average pairwise expression ratios (M) of the 12 reference genes were evaluated using software geNorm. M value is negatively correlated with gene stability, and below 1.5 is considered to be stable expression (Hellemans et al., 2007). As determined by the geNorm, *ACT101*, and *HIS* were the most stable reference genes in total samples. In contrast, *TUA* and *18s* were the least stable reference genes. Under each subset of the treatment, the two best reference genes in drought stress were *ACT101* and *HIS* with the lowest M value. The most preferred genes for normalization in the cold stress were *ACT7* and *HIS*. As for the salt stress, *GAPDH2* and *HIS* were the most stable reference genes. In general, *HIS* was the most stable gene in total or single treatment (Figure 2).

**Figure 3:**
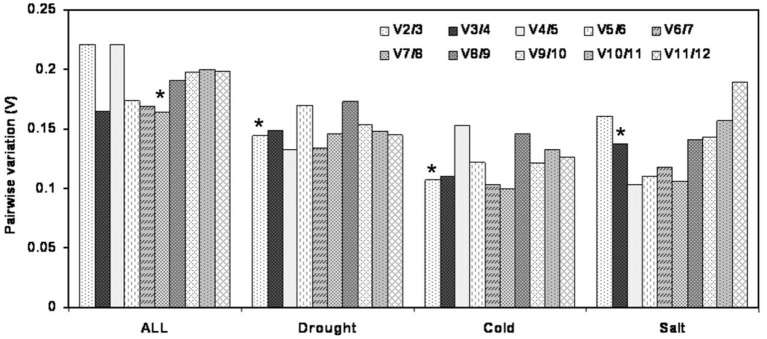
**Pairwise variation (V) of 12 candidate reference genes calculated by geNorm to determine the optimal number of reference genes for accurate normalization**. Asterisk indicates the optimal number of reference genes required for normalization.

The optimal number of the reference genes required for accurate normalization was determined by pairwise variation (Vn/Vn+1). In the subsets of drought and cold stress, the V2/3 value was below 0.15 (0.144 and 0.107, respectively), which suggested that two reference genes should be used for normalization. In the salt treatment subset, three reference genes were sufficient for accurate normalization, as the V3/4 value was lower than 0.15. When total samples were considered, the pairwise variationV7/8 value was the lowest (0.165) but still above 0.15 (Figure 3).

### NormFinder analysis

NormFinder ranks genes based on the stability value for each reference gene. More stable gene expression has the lower stability value. In the subset of drought stress, *TUB* and *ACTB* were the most stable. In cold stress, *HIS* and *ACT101* were the most stable. In salt stress, the top three stably expressed genes were *GAPDH2, ACT101*, and *HIS*. When evaluated the total experimental samples, *ACT7* and *FBA* were the top ranked genes (Table 3). In contrast, *TUA* was the least stable reference gene under drought stress, *ACT11* was the least stable under cold stress and *18S* was the least stably expressed gene under salt stress. The rank in NormFinder was slightly different from that in geNorm. Genes considered as the most stable by geNorm (*ACT101* and *HIS*) ranked third and forth by NormFinder, respectively.

**Table 3:** **Expression stability of the candidate reference genes calculated by NormFinder**.

**Rank**	**ALL**		**Drought**		**Cold**		**Salt**	
	**Gene**	**Stability**	**Gene**	**Stability**	**Gene**	**Stability**	**Gene**	**Stability**
1	*ACT7*	0.602	*TUB*	0.523	*HIS*	0.804	*GAPDH2*	0.520
2	*FBA*	0.619	*ACTB*	0.978	*ACT101*	0.881	*ACT101*	0.582
3	*ACT101*	0.809	*ACT7*	1.111	*ACTB*	0.915	*HIS*	0.591
4	*HIS*	0.857	*MET*	1.270	*ACT7*	1.287	*TUB*	0.936
5	*ACTB*	0.925	*ACT101*	1.290	*GAPDH2*	1.422	*ACTB*	1.039
6	*TUB*	1.006	*FBA*	1.299	*FBA*	1.477	*ACT7*	1.232
7	*GAPDH2*	1.133	*GAPDH2*	1.460	*TUB*	1.607	*TUA*	1.539
8	*ACT11*	1.347	*HIS*	1.582	*GAPDH1*	1.719	*FBA*	1.778
9	*MET*	1.529	*GAPDH1*	1.887	*MET*	1.752	*ACT11*	1.858
10	*GAPDH1*	1.947	*ACT11*	1.919	*18S*	1.790	*MET*	1.880
11	*TUA*	2.079	*18S*	1.933	*TUA*	2.054	*GAPDH1*	2.313
12	*18S*	2.360	*TUA*	2.239	*ACT11*	2.262	*18S*	2.551

*Lower expression stability value indicates more stable expression*.

### BestKeeper analysis

The BestKeeper program determines the stability ranking of the reference genes based on the CV ± SD values. In the drought stress set, *HIS* (2.17 ± 0.51) and *ACT101* (2.55 ± 0.67) with lowest CV ± SD values were identified as stable genes, which were consistent with the results of geNorm. In the cold stress set, only *ACT7* and *HIS* showed SD < 1, which were considered as the most stable genes. In the salt stress treatment, *HIS* (2.24 ± 0.51), *GAPDH2* (3.13 ± 0.85), and *ACTB* (3.26 ± 0.90) were identified as the best reference genes for normalization (Table 4).

**Table 4:** **Expression stability of 12 candidate reference genes calculated by BestKeeper**.

**Rank**	**ALL**			**Drought**			**Cold**			**Salt**		
	**Gene**	**SD**	**CV**	**Gene**	**SD**	**CV**	**Gene**	**SD**	**CV**	**Gene**	**SD**	**CV**
1	*HIS*	0.68	2.91	*HIS*	0.51	2.17	*HIS*	0.53	2.27	*HIS*	0.51	2.24
2	*ACT101*	0.71	2.72	*ACT101*	0.67	2.55	*ACT7*	0.97	4.10	*GAPDH2*	0.85	3.13
3	*FBA*	0.82	2.95	*ACT7*	1.00	4.01	*GAPDH2*	1.10	3.92	*ACTB*	0.90	3.26
4	*ACT7*	0.91	3.89	*TUB*	1.11	4.05	*FBA*	1.11	3.90	*ACT101*	0.94	3.73
5	*TUB*	1.36	4.94	*FBA*	1.17	4.09	*MET*	1.12	3.73	*MET*	1.05	3.52
6	*GAPDH2*	1.42	4.99	*TUA*	1.31	4.60	*ACT101*	1.19	4.76	*FBA*	1.08	3.76
7	*ACTB*	1.42	4.94	*ACTB*	1.53	5.36	*TUB*	1.22	4.50	*ACT7*	1.33	5.66
8	*MET*	1.52	5.02	*MET*	1.80	5.97	*ACTB*	1.64	5.99	*TUB*	1.54	5.95
9	*ACT11*	1.58	5.55	*GAPDH2*	2.00	7.30	*18S*	1.91	13.85	*TUA*	1.67	5.99
10	*18S*	1.86	11.67	*GAPDH1*	2.24	7.91	*TUA*	2.03	7.40	*ACT11*	2.21	7.88
11	*TUA*	1.96	7.20	*18S*	2.31	13.78	*GAPDH1*	2.22	8.29	*GAPDH1*	2.59	10.33
12	*GAPDH1*	2.55	9.54	*ACT11*	2.40	7.76	*ACT11*	2.61	9.20	*18S*	3.17	23.74

### Reference gene validation

**Figure 4:**
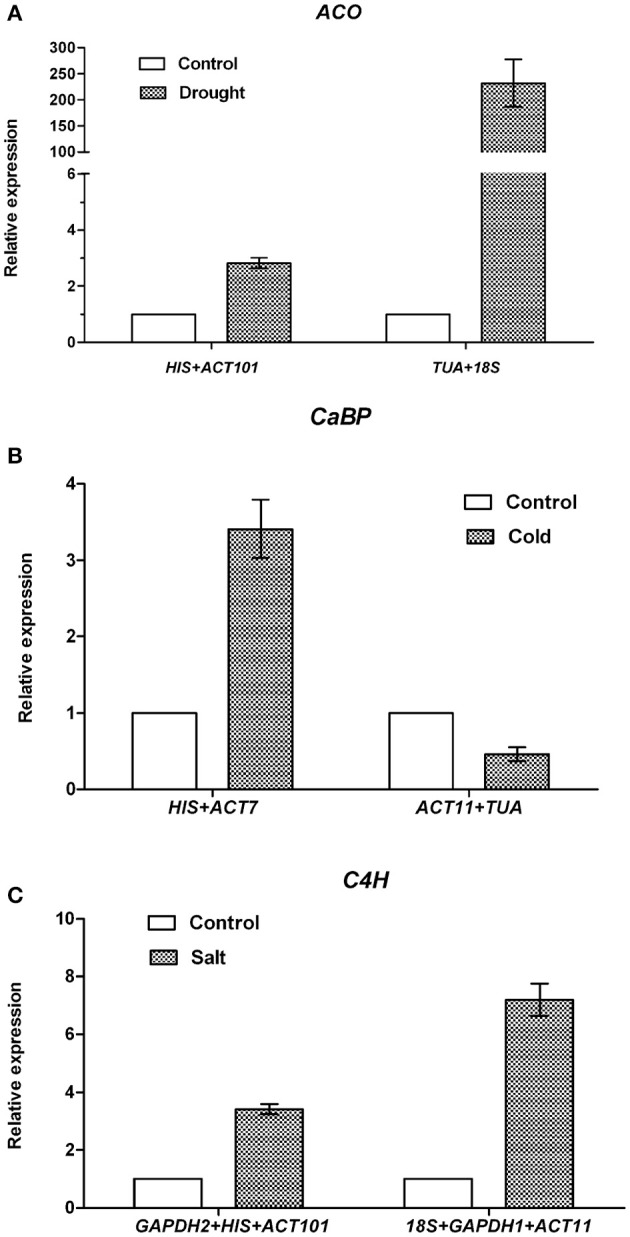
**Normalized expression level of *ACO, CaBP* and *C4H***. **(A)** Relative quantification of *ACO* expression using by the best stable genes (*ACT101* and *HIS)*, and the least stable genes (*TUA* and *18S*) under drought treatment. **(B)** Relative quantification of *CaBP* expression by the best stable genes (*HIS* and *ACT7)*, and the least stable genes (*ACT11* and *TUA*) under cold treatment. **(C)** Relative quantification of *C4H* expression by the best stable genes (*GAPDH2*, *HIS*, and *ACT101)*, and the least stable genes (*18S, GAPDH1*, and *ACT11*) under salt treatment. The average Ct value was calculated from three biological and technical replicates and used for relative expression analyses. Error bars indicate standard errors.

Data generated by the three algorithms across different experimental sets were further compared (Table 5). The best and worst ranked candidate reference genes were selected for normalizing three target genes *ACO*, *CaBP*, and *C4H* under different experimental conditions. Under drought treatment, the expression level of *ACO* was up-regulated 2.8-fold when normalized using the two stable genes (*HIS* and *ACT101*), while the expression level was overestimated (232-fold) when normalized using the least stable combination (*TUA* and *18S*). When *HIS* and *ACT101* were used as the reference genes, the expression level of *ACO* was generally identified with the expression profile in RNA-seq (Figure S2). In the same way, under cold stress, the expression level of *CaBP* was 3.4-fold higher than control when using the most stable reference genes (*ACT7* and *HIS*) as the internal control. By contrast, the expression pattern of *CaBP* was down-regulated when the least stable genes were used (*ACT11* and *TUA*). Based on the result of geNorm, three reference genes were needed for normalization under salt stress condition. The stable gene combination (*GAPDH2, HIS*, and *ACT101*) was used to analyze the transcript abundance of *C4H* under salt stress. *C4H* was 3.4-fold up-regulated evaluated by the stable genes and there was a bias when the worst genes *(18S+GAPDH1+ACT11*) were used for normalization (Figure 4).

**Table 5:** **Expression stability ranking of the 12 candidate reference genes**.

**Method**	**1**	**2**	**3**	**4**	**5**	**6**	**7**	**8**	**9**	**10**	**11**	**12**
**A. RANKING ORDER UNDER ALL STRESS (BETTER–GOOD–AVERAGE)**												
BestKeeper	*HIS*	*ACT101*	*FBA*	*ACT7*	*TUB*	*GAPDH2*	*ACTB*	*MET*	*ACT11*	*18S*	*TUA*	*GAPDH1*
NormFinder	*ACT7*	*FBA*	*ACT101*	*HIS*	*ACTB*	*TUB*	*GAPDH2*	*ACT11*	*MET*	*GAPDH1*	*TUA*	*18S*
geNorm	*ACT101/HIS*		*ACT7*	*FBA*	*TUB*	*ACTB*	*GAPDH2*	*MET*	*ACT11*	*GAPDH1*	*TUA*	*18S*
Comprehensive ranking	*ACT7*	*HIS*	*ACT101*	*FBA*	*TUB*	*ACTB*	*GAPDH2*	*MET*	*ACT11*	*GAPDH1*	*TUA*	*18S*
**B. RANKING ORDER UNDER DROUGHT STRESS (BETTER–GOOD–AVERAGE)**												
BestKeeper	*HIS*	*ACT101*	*ACT7*	*TUB*	*FBA*	*TUA*	*ACTB*	*MET*	*GAPDH2*	*GAPDH1*	*18S*	*ACT11*
NormFinder	*TUB*	*ACTB*	*ACT7*	*MET*	*ACT101*	*FBA*	*GAPDH2*	*HIS*	*GAPDH1*	*ACT11*	*18S*	*TUA*
geNorm	*ACT101/HIS*		*ACT7*	*FBA*	*TUB*	*ACTB*	*MET*	*GAPDH2*	*18S*	*GAPDH1*	*ACT11*	*TUA*
Comprehensive ranking	*HIS*	*ACT101*	*TUB*	*ACT7*	*ACTB*	*FBA*	*MET*	*GAPDH2*	*GAPDH1*	*ACT11*	*18S*	*TUA*
**C. RANKING ORDER UNDER COLD STRESS (BETTER–GOOD–AVERAGE)**												
BestKeeper	*HIS*	*ACT7*	*GAPDH2*	*FBA*	*MET*	*ACT101*	*TUB*	*ACTB*	*18S*	*TUA*	*GAPDH1*	*ACT11*
NormFinder	*HIS*	*ACT101*	*ACTB*	*ACT7*	*GAPDH2*	*FBA*	*TUB*	*GAPDH1*	*MET*	*18S*	*TUA*	*ACT11*
geNorm	*ACT7/HIS*		*FBA*	*MET*	*ACTB*	*GAPDH2*	*ACT101*	*TUB*	*GAPDH1*	*18S*	*TUA*	*ACT11*
Comprehensive ranking	*HIS*	*ACT7*	*ACT101*	*FBA*	*ACTB*	*GAPDH2*	*MET*	*TUB*	*GAPDH1*	*18S*	*TUA*	*ACT11*
**D. RANKING ORDER UNDER SALT STRESS (BETTER–GOOD–AVERAGE)**												
BestKeeper	*HIS*	*GAPDH2*	*ACTB*	*ACT101*	*MET*	*FBA*	*ACT7*	*TUB*	*TUA*	*ACT11*	*GAPDH1*	*18S*
NormFinder	*GAPDH2*	*ACT101*	*HIS*	*TUB*	*ACTB*	*ACT7*	*TUA*	*FBA*	*ACT11*	*MET*	*GAPDH1*	*18S*
geNorm	*GAPDH2/HIS*		*ACT101*	*ACTB*	*ACT7*	*FBA*	*MET*	*TUA*	*TUB*	*ACT11*	*GAPDH1*	*18S*
Comprehensive ranking	*GAPDH2*	*HIS*	*ACT101*	*ACTB*	*ACT7*	*TUB*	*FBA*	*MET*	*TUA*	*ACT11*	*GAPDH1*	*18S*

## Discussion

An accurate and careful expression analysis of stress related genes can bring valid information to reveal more details about the transcriptional networks in stress response process. Therefore, it is required that any transcriptional study be based on accurate selection of suitable reference genes for normalization to ensure the accuracy of the analysis. To date, few studies have undertaken a comparison and selection of reference genes in *O. ochrocephala* or even in any *Oxytropis* species. As a result, this hinders relevant work in characterization of genes involved in stress responses in *O. ochrocephala*. We had performed the first large-scale transcriptome data for *O. ochrocephala* (unpublished), consisting of 118,596 unigenes, which served as the source of the reference gene selection. The study demonstrated that the RNA-Seq data are useful source for candidate reference genes screening and represented an important strategy for large-scale reference gene selection for non-model plants. By using three different computer programs, the 12 candidate reference genes exhibited various performance in their stability in *O. ochrocephala*.

Amongst protein-coding genes, *HIS* was indicated as a good potential internal control under drought treatment. This result was consistent with a previous study in strawberry, which confirmed that *HIS* gene was the most stable reference genes under osmotic stresses and salt stress (Galli et al., 2015). However, in liverwort, as evaluated by geNorm and NormFinder, *HIS* gene showed poor expression stability except for the group involving hormone treatment (Saint-Marcoux et al., 2015). The result demonstrates that in specific species reference genes are regulated differently, each species may have its own stably expressed genes which should be determined for each subset of experimental conditions. *MET* and *FBA* were relatively uncommon reference genes compared with the frequently used ones (commonly used). Several reports have indicated that the expression levels of traditional reference genes vary considerably and the newly discovered reference genes perform better than the traditional reference genes in Arabidopsis (Dekkers et al., 2012), *Brassica napus* L. (Yang et al., 2014b), and tea (Hao et al., 2014). While in this study, neither *FBA* nor *MET* ranked the top three most stable genes under tested experimental conditions. The commonly used reference genes outperformed the novel ones in *O. ochrocephala*. Moreover, based on transcriptome datasets, more novel and stable reference gene could be identified from other *O. ochrocephala* samples in the further study.

The actin gene(s), the most frequently used reference gene (Zhong et al., 2011; Galli et al., 2013), also showed poor consistency in *Brassica napus* L, soybean and peach (Tong et al., 2009; Ma et al., 2013; Yang et al., 2014b). It is notable that among the four actin genes tested in our study, *ACT101* and *ACT2/7* ranked head of *ACTB* and *ACT11*. Similarly, gene expression levels varied between the homologous genes *TUA* and *TUB*, and between *GAPDH1* and *GAPDH2*. In the present study, the commonly used reference gene *TUA* and *GAPDH1* displayed variable expression pattern but *TUB* and *GAPDH2* were more stable in each subset. This result was consistent with an earlier studies in soybean, in that the expression of *Actin2/7* was also more stable than *Actin11* in different tissues (Jian et al., 2008) and similarly in rice, *UBQ5* was the most stably expressed, whereas *UBQ10* exhibited the least stable expression in a given set of tissue samples (Jain et al., 2006). These results suggest that the expression pattern and stability of members from the same gene family may be variable in the same experiment. For these homolog genes, the fluctuated Ct values suggest different levels of expression and they have the same CDS (coding sequence) from different gene loci. This highlights the need to ensure primer specificity at the outset of any gene expression experiment because of the similarity of coding sequence in the genes within the same gene family. In our experiment, primer sets were designed in the UTR to avoid the conserved domain, which enabled the gene specific amplification (Table S2).

Our results indicated that *18S* was the least stable reference gene in almost all the experimental condition. The difference of Ct between *18S* and *HIS* (stable gene) was 7, suggesting over 20-fold of gene expression between the two genes. When *18S* was validated as a reference gene for normalization the target genes *ACO*, the expression pattern was obviously overestimated, consistent with findings in broomrape (González-Verdejo et al., 2008) and rice (Bevitori et al., 2014). According to Jain et al. (2006), the reason why *18S* is not suitable for normalization in real-time PCR analysis is the excessive high expression level compared with the target genes, which make it difficult to analyze the qRT-PCR data by subtracting the baseline. Nonetheless, in the absence of better choices, *18S* is still frequently used as the reference genes for normalizing gene expression because it is independent of developmental stages and external stimuli (Gantasala et al., 2013; Huang et al., 2014).

In order to avoid the erroneous data that may be triggered by using single reference gene, normalization with multiple reference genes is becoming the common way. To evaluate the optimal number of genes required for normalization, geNorm showed V7/8 was the lowest value in total treatment samples. However, using seven reference genes to normalize is not feasible in practice. The V value (pairwise variation) threshold 0.15 should not be considered as an absolute value but rather a suggested one. Several reports have even reported higher V values in some species (De Ketelaere et al., 2006; Wan et al., 2010) and it is dependent on the consideration of the research purpose. Vandesompele et al. (2002) proposed that at least three genes are required for more reliable normalization. Considering these results, three top ranked reference genes *HIS*, *ACT7*, and *ACT101* were appropriate for gene expression normalization under drought, cold and salt conditions. Besides, using different reference genes may lead to opposite or misunderstanding results in normalization of target genes.

To conclude, the current work presented here shows that the transcriptome data provide an effective approach to identify a set of reference genes and the reference genes, *HIS*, *ACT7*, and *ACT101*, were stably expressed under abiotic stress treatments, which should facilitate future studies on gene expression in *O. ochrocephala*.

## Author contributions

Designed and performed and the experiments: HZ, LW; Analyzed data and wrote the paper: HZ, YF; Conducted the experiments: YF, WH; Contributed reagents/materials/fund support: WH, YW.

## Conflict of interest statement

The authors declare that the research was conducted in the absence of any commercial or financial relationships that could be construed as a potential conflict of interest.
