# Supplementary material for: Selection of appropriate reference genes for quantitative real-time PCR in Oxytropis ochrocephala Bunge using transcriptome datasets under abiotic stress treatments
Source: Front Plant Sci. 2015 Jun 30;6:475. doi: 10.3389/fpls.2015.00475 (PMC4484982; doi:10.3389/fpls.2015.00475)
Supplement: Supplementary file 1 [file DataSheet1.DOCX]

**Supporting Information:**


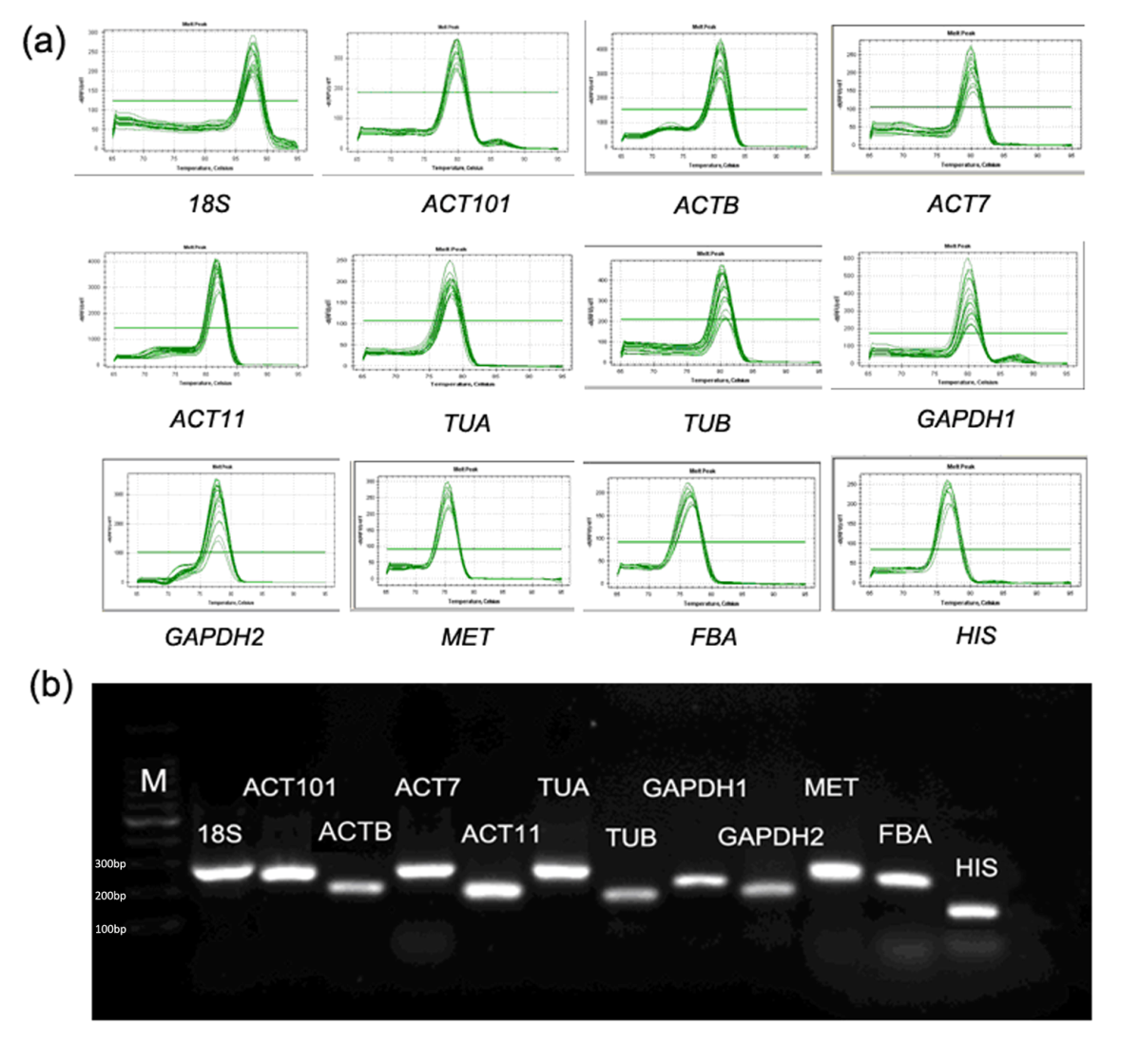


**Figure S1 Melting curves and agarose gel electrophoresis of PCR products**

Specificity of qRT-PCR amplification

(a) Melting curves of 12 reference genes showing single peaks.
(b) Agarose gel (2 %) electrophoresis showing amplification of a single PCR product of the expected size. M represents 100 bp DNA marker.


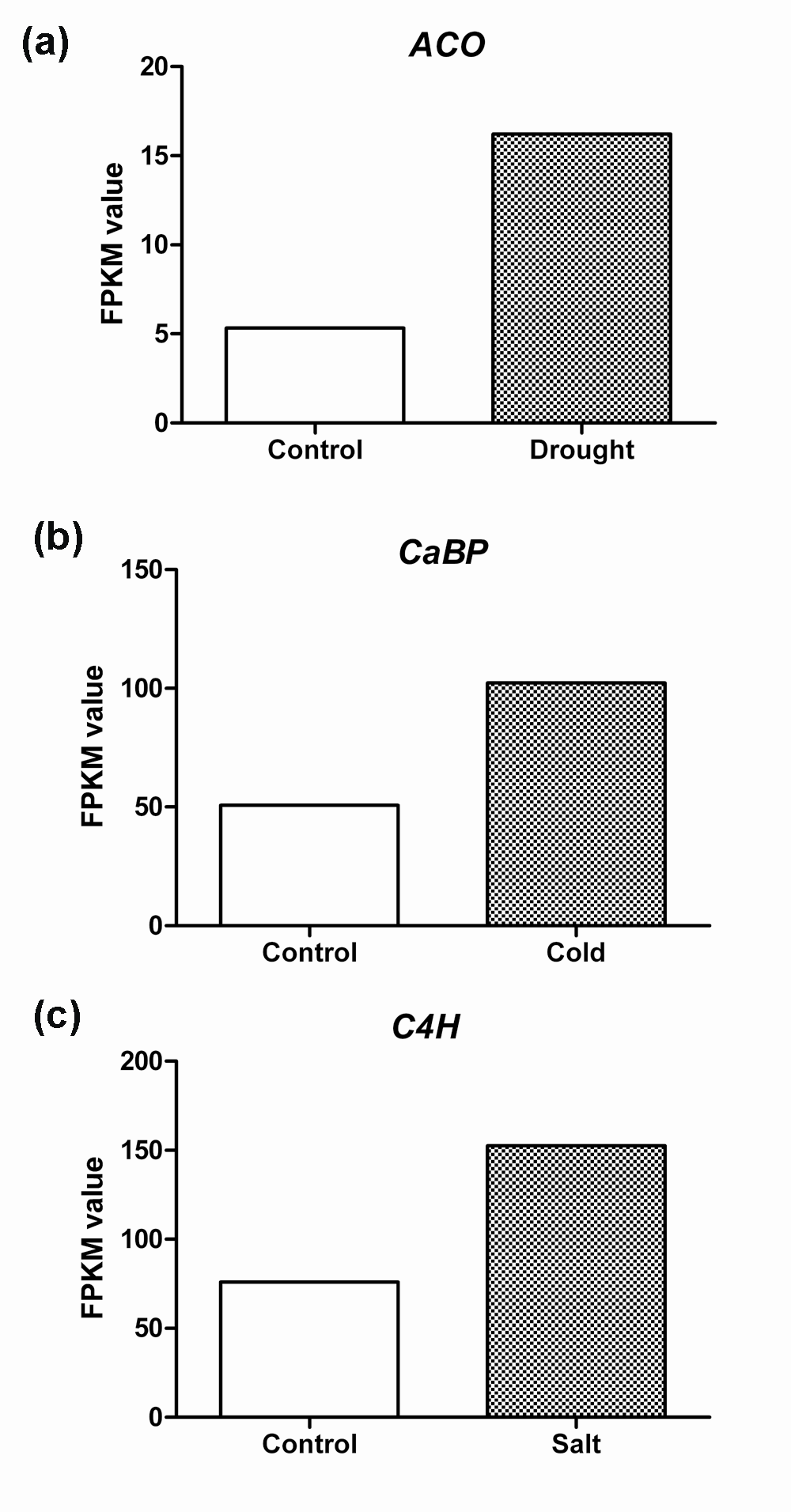


**Figure S2 The expression profiles of the three target genes in RNA-seq**

1. The expression level of *ACO* under control and drought treatment.
2. The expression level of *CaBP* under control and cold treatment.
3. The expression level of *C4H* under control and salt treatment.

FPKM values: fragments Kilobase of exon model per millon mapped reads

**Table S1: Amino acid and nucleotide identity among homologous genes tested in the qRT-PCR-amplified fragment.**

| **Homologous Gene 1** | **Homologous Gene 2** | **Amino acid**  **% / residue** | **Nucleotide Similarity %** |
| --- | --- | --- | --- |
| comp81236_c0 ACT101 | comp82568_c0 ACTB | 24.80 | 39.78 |
| comp81236_c0 ACT101 | comp79791_c0 ACT7 | 25.61 | 50.28 |
| comp81236_c0 ACT101 | comp81236_c3 ACT11 | 26.62 | 39.44 |
| comp82568_c0 ACTB | comp79791_c0 ACT7 | 24.00 | 39.13 |
| comp82568_c0 ACTB | comp81236_c3 ACT11 | 29.32 | 36.97 |
| comp79791_c0 ACT7 | comp81236_c3 ACT11 | 25.84 | 38.1 |
| comp80981_c0 TUA | comp49473_c0 TUB | 28.80 | 39.25 |
| comp83553_c0 GAPDH 1 | comp90007_c0 GAPDH 2 | 28.53 | 35.2 |

**Table S2: Location of the primers in relation to cDNA contig sequences.**

| **Gene symbol** | **Gene ID** | **Gene length (bp)** | **ORF** | **Conserved Domain** | **Primer Location** |
| --- | --- | --- | --- | --- | --- |
| *18S* | comp82308_c0 | 7829 | 2738-3376 | No conserved domains | 178-459 |
| *ACT101* | comp81236_c0 | 1849 | 139-1272 | 157-1269 | 1221-1452 |
| *ACTB* | comp82568_c0 | 732 | 70-732 | 1-732 | 531-716 |
| *ACT7* | comp79791_c0 | 1936 | 281-1414 | 293-1411 | 1278-1543 |
| *ACT11* | comp81236_c3 | 1167 | 139-1167 | 151-1167 | 86-253 |
| *TUA* | comp80981_c0 | 1970 | 297-1646 | 297-1592 | 1583-1842 |
| *TUB* | comp49473_c0 | 1969 | 280-1629 | 283-1542 | 1710-1897 |
| *GAPDH1* | comp83553_c0 | 2197 | 912-1865 | 765-1766 | 548-758 |
| *GAPDH2* | comp90007_c0 | 1731 | 656-1420 | 674-1408 | 465-626 |
| *MET* | comp71233_c0 | 661 | 49-249 | 121-243 | 334-584 |
| *FBA* | comp67830_c0 | 1851 | 96-1295 | 96-1292 | 1483-1707 |
| *HIS* | comp67746_c0 | 955 | 84-494 | 84-464 | 500-594 |

**Table S3 Information of selected target genes for validation**

| **Gene ID** | **Gene symbol** | **Accession number** | **Gene length (bp)** | **NR description** | **Forward Primer**  **Sequence [5'-3']** | **Reverse Primer**  **Sequence [5'-3']** |
| --- | --- | --- | --- | --- | --- | --- |
| comp81166_c0 | *ACO* | KR822237 | 124 | aminocyclopropane-1-carboxylate oxidase | TGATTATTTAGAGCTTTATGGGAAG | CCGAAACAAAGGTATTAGGTTAC |
| comp49552_c0 | *CaBP* | KR822235 | 158 | Calcium binding protein | TGTCCACACTTCTTGATGATATAGA | ATCCATCATTCATCATACCCAC |
| comp76428_c0 | *C4H* | KR822236 | 208 | cinnamate 4-hydroxylase | CCACCAACATTTCATTTCC | CTTACCGAAGATTGGAACG |
